# The Complex Interplay of Inflammation, Metabolism, Epigenetics, and Sex in Calcific Disease of the Aortic Valve

**DOI:** 10.3389/fcvm.2021.791646

**Published:** 2022-01-06

**Authors:** Silvia Ferrari, Maurizio Pesce

**Affiliations:** Unità di Ingegneria Tissutale Cardiovascolare, Centro Cardiologico Monzino, IRCCS, Milan, Italy

**Keywords:** calcific aortic valve disease (CAVD), epigenetics, metabolism, inflammation, sex

## Abstract

Calcification of the aortic valve is one of the most rapidly increasing pathologies in the aging population worldwide. Traditionally associated to cardiovascular risk conditions, this pathology is still relatively unaddressed on a molecular/cellular standpoint and there are no available treatments to retard its progression unless valve substitution. In this review, we will describe some of the most involved inflammatory players, the metabolic changes that may be responsible of epigenetic modifications and the gender-related differences in the onset of the disease. A better understanding of these aspects and their integration into a unique pathophysiology context is relevant to improve current therapies and patients management.

## Introduction

Calcification of cardiac valves is one of the most rapidly increasing pathologies in the aging population worldwide. Among these, calcific aortic valve disease (CAVD) predominates and it is reaching epidemic proportions, with approximately one-third of the individuals over 65 presenting sub-clinical evidence of the pathology, which would result in severe impairment of the valve function (1). Since life expectancy has increased over the last decades and the incidence of degenerative valve disease increases with age, it becomes more and more urgent to define the underlying pathophysiological mechanisms to improve current therapies that up to date consist predominantly of valve substitution (2). The disease caused by calcification of the aortic valve is often associated to several risk conditions, also typical of other cardiovascular disorders such as coronary artery disease and atherosclerosis (3). Indeed, several studies revealed a strong association between the presence of severe aortic stenosis (AS) and atherosclerosis (4), despite other data suggest that the pathology might be the consequence of independent mechanisms (5).

Calcific aortic valve disease is characterized by an obstruction of the outflow, often accompanied by regurgitation, and it has chronic features. In fact, it progresses gradually with an increasing structural deterioration, which results in an impairment of ventricular function, and ends in heart failure (6, 7). Although the mortality rate does not increase when stenosis is still asymptomatic, it exceeds 50% 2 years after the onset of symptoms if not surgically treated (8), and currently there are no effective therapies to prevent or retard its progression (9, 10).

Aortic stenosis begins with local structural changes of the extracellular matrix (ECM) in the leaflets, and evolves into stenosis with the accumulation of calcium deposits, which distort the geometry of the valve, initially without, or with just mild clinical symptoms (9). Anatomical, genetic, and clinical factors contribute to the pathogenesis of the disease that progresses throughout different phases (8). The first sign of valve degeneration is characterized by the stiffening of the leaflets (the so called sclerotic phase) caused by over deposition of ECM; this phase precedes a progressive formation of calcium nodules (also along with new vessels), initially in the proximity of the branching onto the aortic ring, and a thereafter in the entire cusps. The first macroscopic calcium deposits accumulate in the central portion (the so-called “belly”) of the cusps and the commissures between the adjacent leaflets, predominantly in the fibrosa. Since this portion is characterized by the presence of large collagen fibers that maintain the structural integrity of the leaflets and are deputed to resist to the compression forces occurring at the valve closure, it has been speculated that the differentiation into bone-like cells has also a major mechanical component (11). The large calcific nodules deposited in the cusps finally protrude on the aortic side, and interfere with the opening of the valve compromising valvular function (12). The progression from sclerosis to obstruction occurs in about 10–15% of subjects in a period between 2 and 5 years (8). In presence of moderate valve obstructions, progressive stenosis occurs almost in every patient, many of whom have to undergo valve replacement (8). In fact, progressive restriction of the valve orifice causes further mechanical stress, and this creates the conditions for rapid evolution toward a severe stenosis. When the orifice measures (aortic valve area) <1 cm^2^ (*vs*. the normal 2.5–4.5 cm^2^), the valve generates a pressure gradient of over 40 mmHg providing an indication to surgical replacement of the valve (13).

Age, sex, smoking, hypercholesterolemia, and hypertension are generally considered to be implicated in aortic valve stenosis; however growing evidence suggest that other factors cooperate to disease pathophysiology, especially during the transition from the slow sclerotic phase to the rapid stenotic progression (12). In this contribution, we will provide an overview on existing factors and some emerging mechanisms that are increasingly connected to the disease such as such as epigenetics, metabolic alterations, and sex, in the perspective of a better comprehensive vision of the pathology as a whole.

## From Inflammation To Valve Degeneration: The Main Players

One of the first events triggering CAVD is the damage of the endothelium on both sides of the leaflets, resulting from local alterations of blood shear stress. The endothelial damage allows the infiltration of lipids such as low-density lipoproteins (LDL), which become oxidized (ox-LDL) and activate an inflammatory response (14–16). The inflammatory response is characterized by the activation of endothelial cells with increased expression of adhesion molecules, such as vascular cell adhesion molecule 1 (VCAM1) and intercellular adhesion molecule 1 (ICAM-1), responsible for the recruitment of monocytes/macrophages and T cells (17). These cells secrete inflammatory cytokines, including transforming growth factor beta 1 (TGF-β1), a critical pro-fibrotic growth factor involved in myo-fibroblast differentiation of the valve interstitial cells (VICs) (13), the cells deputed professionally to repair the ECM in the tissue continuously subjected to mechanical stress (18). Tumor necrosis factor alpha (TNF-α) is an important mediator of inflammation that is also involved in calcification (13), in cooperation with other factors such as interleukins (IL) (e.g., IL-1β, IL-2, and IL-6) (14–16). The involvement of inflammation in CAVD onset is confirmed by the presence of lymphocytes and macrophages together with the osteoblast-like cells nearby the calcific areas (16). In particular, immunohistochemistry analyses revealed chronic inflammatory infiltrates, composed by CD68^+^ macrophages, CD45^+^ leukocytes, and CD3^+^ T cells on degenerated valve leaflets. Intriguingly, the density of leukocytes clusters correlates with the progression rate of AS (19). The association between inflammation and valve calcification was recently confirmed *in vivo* using 18F-fluorodeoxyglucose positron emission tomography in patients with severe CAVD showing a high amount of inflammatory infiltrates compared to mild CAVD patients (20).

Central in pathological progression of the valve is the pathway controlled by nuclear factor (NF)-κB, one of the most known transcriptional factor involved in inflammatory response that upon activation by proinflammatory cytokines translocates into the nucleus directly activating gene transcription [e.g., IL-6, CCL-5, CXCL-11, BCL-2, BAX (21–23)] and controlling cell survival, proliferation and innate immunity responses. The NF-κB canonic pathway is activated by TNF-α, IL-1β, and ROS-induced cellular stress (24). IL-6 is a cytokine secreted by different cells type such as macrophages and lymphocytes and has been reported to be highly expressed in valves with severe calcification (25). Husseini and colleagues demonstrated that IL-6 promotes the expression of Bone Morphogenic Protein (BMP-2), known to be responsible of valve leaflets calcification (25). Moreover, IL-6 silencing by the use of siRNA resulted in a decrease in human VICs calcium deposition and, the treatment with an inhibitor of NF-κB exerted the same effect. Another cytokine that plays a central role in valve calcification process is IL-1β. Several studies showed that IL-1β levels are significantly higher in stenotic valves. Similarly to the mechanism described for IL-6, the pharmacological treatment with an inhibitor of NF-κB determines a decrease of both IL-1β secretion and calcium deposition (26). Moreover, it has been demonstrated that IL-1β promotes the expression of MMPs, which degrade the ECM and promote the activation of latent TGF-β (27), thus contributing to worsening the valve stenosis (28).

Isoda and colleagues showed that IL-1 receptor agonist (IL-1Ra) exerts a beneficial effect in the context of valve degeneration: IL-1Ra-induced deficiency in mice inflammatory cells resulted in AS and increased inflammation response (29). IL-37, an anti-inflammatory member of the IL-1 family, has been reported to play a protective role by decreasing expression level of BMP-2 and, thus, interfering with calcifying mechanisms. Zeng et al., indeed, not only found that recombinant IL-37 suppresses the osteogenic responses in human VICs, but also reduced valve thickening in mice subjected to high fat diet (30).

A particularly interesting role in calcification of the aortic valve is that of the Cyclooxygenase-2 (COX-2) enzyme, a crucial component of the arachidonic acid metabolism involved in the synthesis of prostaglandins and thromboxane (31). The function of COX-2 in cardiovascular disease and valve calcification appears controversial. In fact, inhibition of the enzyme by celecoxib, along with genetic depletion in the Klotho deficient mice (a known model of valve calcification) reduced calcification of the valve and of animal-derived VICs *in vitro* (32). On the other hand, a word of caution about the therapeutic potential of COX-2 inhibition by the celecoxib has been also raised by other studies, showing that in human VICs from stenotic valves treatment with the drug may increase the so-called dystrophic calcification, a process mediated by TGF-β1 and related to VICs myofibroblast differentiation and apoptosis (27, 33–36). Early histological studies on 347 surgically excised aortic valves showed that 83% of them presented dystrophic calcifications, while lamellar bone with active bone remodeling were observed in only 13% (37). Although further investigations are needed, these data suggests that dystrophic calcification may play a crucial role in the progression of the pathology.

Another event crucial for the initial stages of the valve calcification is the direct participation of valve-resident cells to leaflets remodeling through phenotype switching into matrix remodeling cells. For example, the endothelial cells covering the leaflets undergo endothelium–mesenchymal transition (EndMT), and progressively acquire the phenotypic/functional characteristics of mesenchymal cells, expressing both endothelial and mesenchymal cell markers such as N-cadherin and α-smooth muscle actin (αSMA). Since mesenchymal phenotype is defined by enhanced cell contractility (38) and enhanced collagen production (39), EndMT contributes to increase the pool of cells potentially participating to valve tissue remodeling. In addition to inflammatory stimuli, the excess of shear stress occurring locally on the cells promotes this phenotypic transition. Mahler et al. developed a 3D model to quantify the effects of shear stress on the EndMT and found that low steady shear stress up-regulated the process as well as the expression of inflammation and tissue remodeling related gene expression (40).

Particularly important for the entire process of valve pathologic progression is the role of the VICs. Initially classified as simple fibroblasts fulfilling the matrix-renewing activity in the valve continuously subjected to mechanical stress, these cells have been better characterized with classification in various sub-phenotypes and different functions in valve tissue repair and pathologic evolution. Indeed, the current literature describes four different VIC phenotypes with specific functions in normal valve physiology and pathophysiology: stem cell-derived progenitor VICs (pVICs), quiescent VICs (qVICs), activated VICs (aVICs), and osteoblastic VICs (obVICs) (41). While pVICs are a heterogeneous population of progenitor cells involved in tissue repair, qVICs are resting cells that maintain physiologic valve structure and function. Nevertheless, as a consequence of heart valve insults, such as mechanical stimuli or pathological injury, qVICs can become: (i) activated (aVICs), expressing matrix metalloproteinases (MMPs), involved in remodeling of ECM, eventually leading to sclerosis (13, 14, 41, 42); (ii) obVICs, in presence of osteogenic and chondrogenic-promoting factors. obVICs actively participate in the valve calcification process by promoting chondrogenesis and osteogenesis, defining the stenotic stage of the disease (14, 43). At this stage, cells are regulated by Runt related transcription factor 2 (RUNX-2) as well as bone morphogenetic proteins: obVICs secrete several proteins expressed in bone tissue, such as osteopontin, osteocalcin, alkaline phosphatase. Finally, calcium nodules are deposited over the matrix of the valve and organized into structures similar to lamellar bone (37, 44). Although it is controlled by similar molecular pathways, calcification of the valve is, finally, a peculiar process that has characteristics in common with vascular calcification more than with bone formation. In this respect, particularly important was the work by Bertazzo and colleagues showing that the localization of spherical mineralized particles may precede the appearance of mature calcium-depositing cells and the large calcium nodules. By examining the calcific lesions with nano-analytical electron microscopy, they determined that spherical particles are composed of crystalline hydroxyapatite which are different at a crystallographic and structural level from the calcium deposits in the bone (45). These mineralized microparticles may begin to be secreted by VICs before their terminal osteoblast-like differentiation, due to the sensitivity of the cells to matrix mechanics (46). In addition, this modality of calcium deposition was not found only in the valve but also in calcific arteries, showing a peculiarity of the cardiovascular calcification mechanism due to VICs and vascular smooth muscle cells (VSMCs). Besides calcification, various studies have demonstrated the occurrence of neovascularization during CAVD progression (47, 48) and the role played by angiogenic pathways in the pathophysiology of aortic valve disease (49–51). For example, Arevalos and colleagues investigated the function of VICs when cultured under pro-angiogenic conditions, and showed that these cells acquire a pericyte phenotype able to promote endothelial cells motility and angiogenesis (52).

**Figure 1:**
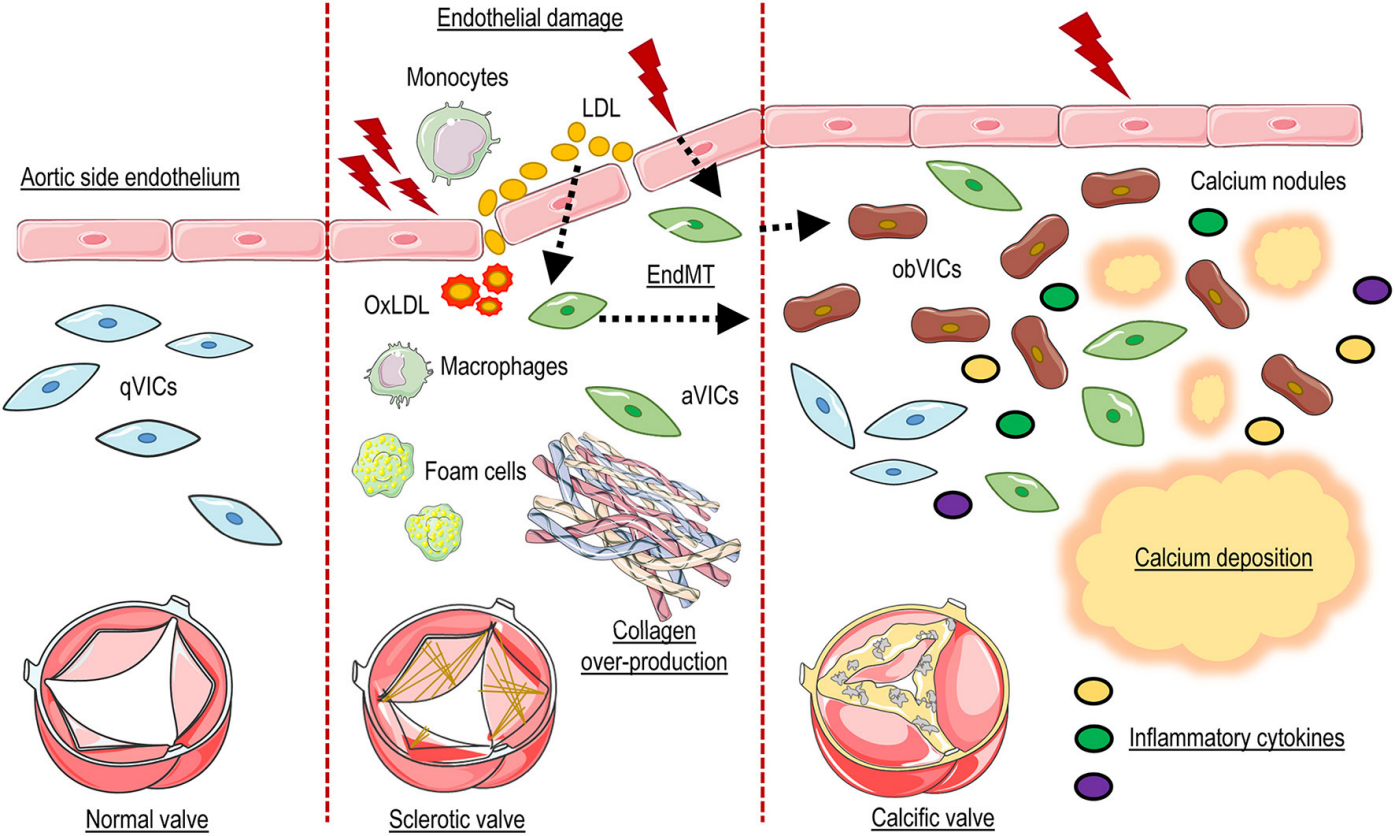
Schematic representation of CAVD pathophysiology. Endothelial damage allows penetration and accumulation of lipids and inflammatory cells into the valve tissues, and End-MT. Oxidative modification of lipoproteins (OxLDL), formation of foam cells, and the production of inflammatory cytokines promotes the activation of resident valve interstitial cells (aVICs) which, in combination with invading inflammatory cells and oxidized lipids, promote fibrosis and thickening of the valve interstitial tissue. In the last phase of the pathology aVICs give rise to calcific cells (obVICs) responsible for secretion of mineralized microparticles and formation of calcium nodules.

Valve interstitial cells, finally, represent also active players in the inflammatory process and are able to directly respond to inflammation. Indeed they express toll-like receptors (TLRs), a class of receptors that initiate the innate immune response, which contributes in the release of inflammatory cytokines (e.g., IL-6, IL-17, IL-1β) by activation of NF-kB pathway (53). TLR-4 and TLR-2 were the first members of the TLR family characterized in VICs and it has been reported an increase of these receptors in calcific valves compared to controls (54). Meng and colleagues described the TLR-dependent activation of NF-κB signaling and the subsequent induction of cytokines and adhesion molecules (55). Indeed, they found that activation of NF-kB promotes the secretion of IL-6 and IL-8, resulting in increased expression of both BMP-2 and RUNX-2 leading to calcium production (56). Interestingly, this effect was abolished by targeting expression of TLR-2 and−4 by a siRNA approach (54) (Figure 1).

## Metabolic Changes, Inflammation, and Biological Implications

It is known that inflammatory responses are also related to relevant changes in metabolic activity inside tissues. This includes high production of reactive oxygen species (ROS), local depletion of nutrients, and increased oxygen consumption (57). Studies demonstrated that these modifications may be caused by inflammatory cells recruitment and proliferation that may give rise to an imbalance in valve tissue oxygen metabolism (58). A recent publication by Tandon et al., using two-photon excited fluorescence (TPEF) microscopy, demonstrated that optical redox ratio (ORR) was associated with phenotypic changes of VICs into osteogenic cells (59). In particular, as it was previously shown in mesenchymal stem cells (60), ORR decreased in porcine VICs induced to differentiate into bone-like cells and this correlated with the expression typical CAVD markers such as RUNX-2 and TGF-β (59). Intriguingly, ORR has been associated to an increase in the glycolytic pathway activation, reflecting levels of oxidative phosphorylation (60). Finally, Wei and colleagues showed that osteoblast differentiation coincided with an increase in glucose uptake (61). Taken together, these data suggest that changes in metabolism associated to osteoblast differentiation of VICs, such as higher levels of glycolysis products (i.e., lactate) and upregulation of specific factors (i.e., VEGF), might be responsible of the angiogenesis associated with the onset of CAVD.

It is known that the early stage of CAVD, defined by the recruitment of immune cells and subsequent sclerosis of the valve leaflets, is characterized by reduced oxygen availability and this activates transcriptional signatures dependent on the hypoxia inducible factor-1 alpha (HIF-1α) and 2α (HIF-2α) activity. It has been reported an increase in HIF-1α and HIF-2α production in stenotic valves and, interestingly, the activity of these factors is promoted by the same cytokines inducing NF-κB (62). In particular, NF-κB and HIF-2α, have been detected in the leaflets from patients with AS, but not in those from with annulo-aortic ectasia, used as controls, where both transcription factors co-localized in cells juxtaposed to the large calcific lesion (63). Further suggesting the roles of *HIF-1* gene products in valve disease is the finding that HIF-1α affects valve phenotype by upregulating the expression of vascular endothelial growth factor (VEGF) and, thus, promoting neo-angiogenesis typical of valve diseases (64, 65). Further supporting the relevance of HIF-1 in control of valve calcification, there is also the evidence that the pharmacological inhibitor of HIF-1α PX478 significantly inhibited calcium deposition caused by endothelial activation resulting from disturbed flow (66). Pharmacological experiments demonstrated the involvement of HIF-1α in the induction of calcification and, intriguingly, the response was more robust in VICs from male donors (67). Since the presence of inflammatory cytokines can also induce the overexpression of VEGF (68), this suggests a convergence of hypoxia- and inflammatory-related pathways intimately connected to valve ECM structural remodeling contributing in VICs activation and calcification.

Activation of hypoxia-related pathways is not the only modality by which metabolic stress can hamper valve disease. In fact, valve calcification depends also on variation of tissue redox balance, and in particular on the release of ROS, i.e., ${\text{O}}_{2}^{-}$, and the concomitant decrease in the expression of the superoxide dismutase (SOD) isoforms—the main enzymatic scavengers of H_2_O_2_. In another study, it was demonstrated that degenerated valves exhibited an uncoupling of the nitric oxide synthase (NOS) and the nitric oxide scavenger (NOX) activity. Intriguingly this uncoupling triggered by inflammation determined a reduction in valve catalase activity and, as a consequence, a higher level of H_2_O_2_ inducing the expression of osteogenic genes such as RUNX-2 (69).

It has been reported that systemic or local inflammation caused by diabetes may contribute to calcium deposition by inducing local inflammation resulting in vascular calcification by glycoxidative modification of lipids in bones and artery walls. In particular, the expression of osteoblastic marker osteocalcin has been detected in endothelial progenitor cells obtained from pre-diabetic patients (70). Moreover, diabetes may contribute to valve degeneration process by vitamin K-dependent activation of Matrix Gla protein (MGP), an extracellular protein with a relevant role in the process of vascular calcification (71). Gla-containing proteins, indeed, were detected in human and porcine calcified aortic valves while none were found in control tissues (72). In a recent study, Choi and colleagues demonstrated the role of insulin-like growth factor-1 (IGF-1) (73) and dipeptidyl peptidase-4 (DPP-4) in valve degeneration (74). Their results showed that nitric oxide (NO) depletion in vascular endothelial cells (VECs) activated NF-κB in VICs. NF-κB induced DPP-4 expression which, interfering with IGF-1, promoted the osteogenic differentiation of VICs. Interestingly, pharmacological inhibition of DPP-4 activity in a rabbit CAVD model resulted in significant improvements in aortic valve function (75). Rattazzi and colleagues investigated the effects of L-Arginine, the main precursor of NO, in VICs osteogenic differentiation. Proteomic and gene expression analysis showed that L-Arginine inhibited inflammatory activation of VICs and prevented osteogenic differentiation reducing matrix calcification (76).

## Epigenetic Regulation Relies on Metabolites Availability

Epigenetics is defined as a series of mitotically and meiotically transmissible DNA/chromatin modifications, which do not affect the primary DNA sequence, but can determine extensive variations in gene expression (77, 78). Epigenetics controls genome functions through different covalent modifications of the chromatin, consisting of DNA methylation and histone modifications, as well as by interactions with non-coding RNA. These modifications change the structure of the chromatin and, consequently, the accessibility of DNA to transcription initiation and elongation factors involved in DNA transcription, replication and repair (79). For the aims of this review, it is necessary to briefly describe the main epigenetic modification: (i) DNA methylation consists of the covalent binding of a methyl group to the 5′ carbon of cytosine and, since it interferes with the binding of or transcription factors DNA-binding proteins on chromatin, it is usually associated with gene expression repression; (ii) histones, the structural component of the chromatin, are subjected to several post-translational modifications such as acetylation that promotes the relaxation of the chromatin and this correlates with gene expression activation, and methylation that can have both effects depending on the target residues and methylation level (80). In particular, hypo-acetylation and hyper-methylation induce chromatin condensation with subsequent gene expression repression (81). It has been reported that changes in RNA/DNA methylation and histone acetylation may have a role in the osteogenic differentiation of VICs. For example it was shown an interesting connection between VICs calcification and N6-methyladenosine (m^6^A) RNA-methylation *via* the upregulation of methyltransferase-like 3 (METTL3) and repression of the helix-loop-helix transcription factor TWIST (82, 83), while in another study, methylation of the *NOTCH-1* promoter was found to cause valve calcification through reduction of the NOTCH intracellular domain (NICD) activity and consequent increase of the Wnt/β-Catenin signaling causing enhanced expression of pro-calcific genes. Particularly interesting is the role of *NOTCH-1* gene product in valve calcification in an epigenetic perspective. Studies of human mutations have indeed shown that haploinsufficiency of *NOTCH-1*, genetically linked to valve calcification (84, 85), determines de-repression of inflammatory and osteogenic genes caused by changes in the levels of lysine 27 acetylation on Histone H3 (86). Another epigenetic mechanism involves NOTCH-1 regulation of valve disease through the role of long-non-coding RNA H19. H19 acts as a suppressor of *NOTCH-1* transcription via P53 (87), and through suppression of myofibroblast VICs phenotype and dystrophic calcification of the cells, especially in the presence of mechanical stimulation (88).

Variations in the histone modification profile have been demonstrated to have a pro-inflammatory and osteogenic role in CAVD development, since they seem to be involved in shear-stress induced pro-inflammatory pathways via alteration of silent information regulator-two (SIRT) histone deacetylates (HDAC) expression (17, 89, 90). Within the epigenetic program, chromatin-modifying enzymes play a central role for regulation of cell-specific transcriptional networks and their activity depends directly on metabolites such as acetyl-coenzyme A, S-adenosylmethionine, and NAD^+^ (91). Since these metabolites act as substrates and/or cofactors for the enzymatic activity of chromatin modifiers, they are essential for coupling chromatin-dependent gene regulation with the metabolic state of the cell. For example, the activity of histone acetyltransferases (HATs) and HDACs is highly dependent on intracellular levels of acetyl-CoA, an intermediary metabolite and substrate for histones acetylation, giving a strong example of the interplay between metabolism and chromatin dynamics (92). Growing evidence demonstrate that the availability of acetyl-CoA is closely associated with histone acetylation, as confirmed by genome-wide analyses from yeast to mammals (93). Moreover, Lee and colleagues demonstrated that non-acetyl histone acylation are more common under conditions of low glucose, a typical condition of hypoxia and consequent inflammation occurring in CAVD (58). Among Sirtuins, deacetylases that target histones and several transcription factors (94), *SIRT-6* has been reported to decrease in aortic valves from AS patients (95). Moreover, in human VICs, the inhibition of *SIRT-6* promotes calcium deposition, inducing the activation of pro-osteogenic pathways and triggering osteogenic differentiation (96). In keeping with these findings, Li and colleagues demonstrated that the activity of the histone acetyltransferase p300 caused an increase in porcine VICs osteoblast-like differentiation (97), while its inhibition resulted in a reduction of Vit D-induced porcine aortic valve calcification (98).

**Figure 2:**
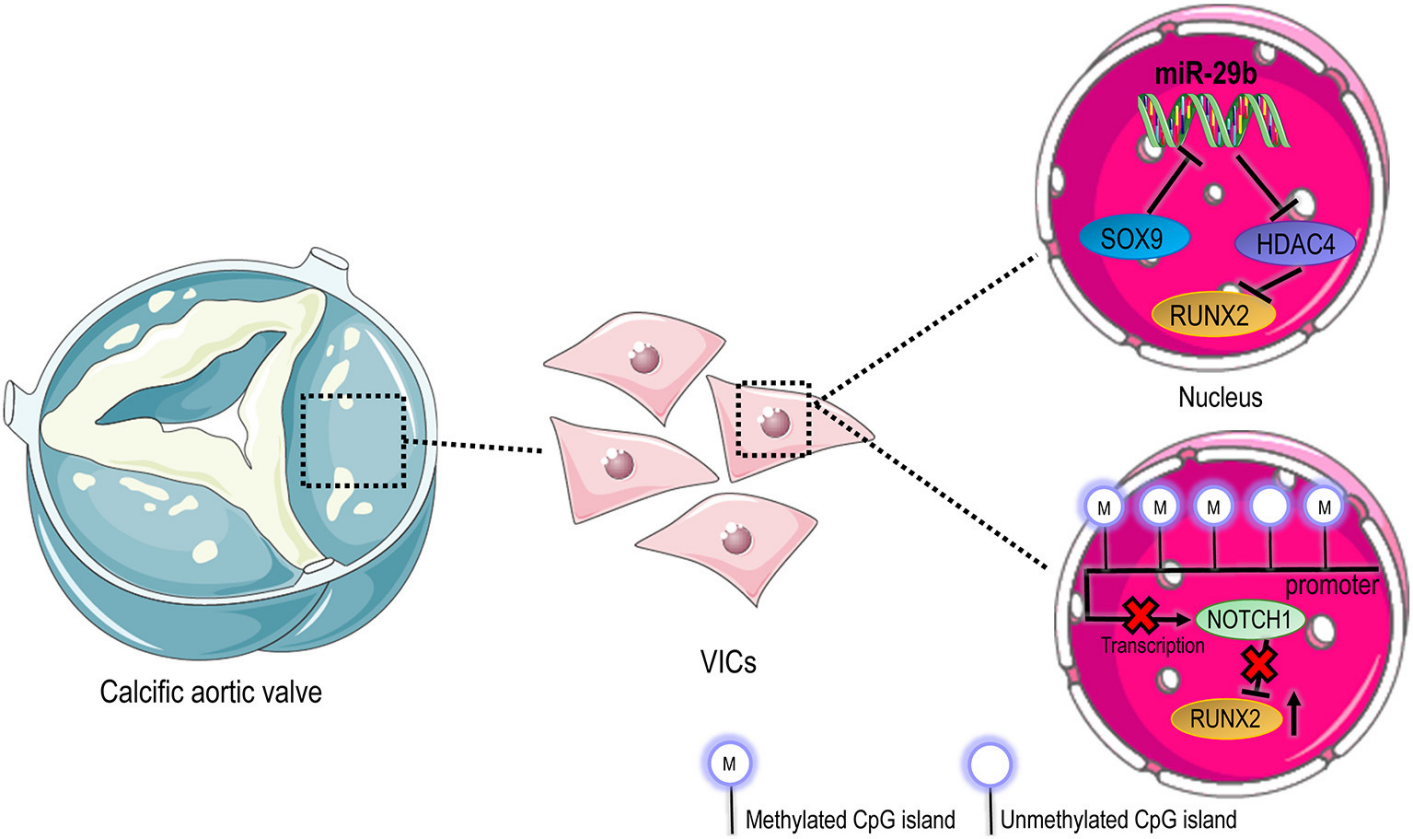
Epigenetic regulation in CAVD. Compared to cells in native valves, where VICs have a prevalent quiescent phenotype, activated and osteoblastic VICs in pathologic valves undergo an extensive molecular reprogramming that involves different modalities of epigenetic control. In the top panel it is represented the transcriptional circuitry that controls the expression of *Runx2* pro-osteogenic transcription factor through regulation of HDAC4. On the bottom of the panel, the downregulation of *Notch1* due to methylation of promoter sequences leads to upregulation of *Runx2*, favoring the osteoblastic differentiation of VICs.

MicroRNAs (miRNAs) are RNA species transcribed but not translated into proteins and can be therefore considered epigenetic regulators (99). To date, several studies demonstrate the involvement of miRNAs in CAVD, implicating potential clinical applications as biomarkers and therapeutic targets. Zheng et al. investigated the role of miR-214 in the inflammatory reaction and the consequent osteoblastic activation of human VICs (100). In particular, performing enzymatic assays, they detected a higher expression level of NF-κB, TLR-4, and miR-214 in the blood and aortic valve tissue samples of patients with CAVD compared to control ones. Furthermore, knockdown of miR-214 was able to inhibit the secretion of IL-6, IL-8, ICAM-1 (100, 101). Up-regulation of human myeloid differentiation factor 88 (MyD88) promoted the secretion of pro-inflammatory factors and increased calcium deposition over the leaflets and increased the expression of RUNX-2 and BMP-2 (100). The levels of miR-29b have been reported to significantly increase during human VICs trans-differentiation into osteoblasts (102). Indeed, the overexpression of this miRNA induced a significant increase of RUNX-2 while its silencing inverted the trend (102). Moreover, miR-29b has been reported to repress transcription of HDAC4, which in turn reduces RUNX-2. MiR-29b acts a positive regulator of RUNX-2 through HDAC-4, then affected the balance of SOX-9/RUNX-2 and finally led to calcification (103). Besides, SOX-9 is a negative regulator of miR-29b in VICs pathological activation: reduced levels of SOX-9 resulted in a upregulation of miR-29b expression and calcification progression (103) (Figure 2).

Clonal hematopoiesis of indeterminate potential (CHIP), defined by the presence of expanded somatic hematopoietic stem cell clones in the bone marrow of aged patients without hematologic malignancy, is a novel risk factor in cardiovascular diseases (104). Being associated to mutations of gene encoding for epigenetic active enzymes related to DNA methylation [e.g., DNA methyltransferase-3α (*DNMT-3A*) and Tet Methylcytosine Dioxygenase-2 (*TET-2*)] (105) or cell cycle control (*P53*) (106), CHIP creates an imbalance in the amount of committed innate immunity cells with a pro-inflammatory phenotype, thus increasing the overall cardiovascular risk due to inflammation and accelerated senescence (106, 107). Although the pathology primarily hampered by CHIP is atherosclerosis, recent evidences suggested that valve disease may be also affected by this new risk condition, as shown in two recent studies reporting the association of DNMT3A and TET2 CHIP-mutations with increased inflammatory cytokines secretion by innate immunity cells, and an increased risk of severe aortic valve stenosis, also connected to poor survival after implantation of transcatheter valve implants (TAVI) (108, 109).

Taken together, these data suggest a close association of epigenetic regulation, inflammatory response, and valve degeneration. Since epigenetic modifications have a crucial impact on cellular functions and most of the epigenetic modifications are reversible, the employment of epigenetic drugs may represent a novel therapeutic approach. For example, the treatment of cardiac mesenchymal cells (CMSC) obtained from type 2 diabetic patients with HAT activator Pentadecylidenemalonate-1b (SPV106) was able to restore proliferation and differentiation to levels observed in control cells from normoglycemic donors (110). Another innovative therapy may consist in the use of epigenetic-active drugs such as HDAC inhibitors (HDACi). There are evidences showing that the pan-HDACi suberoylanilide hydroxamic acid (SAHA) attenuated pathological cardiac remodeling in rodent and rabbit models of myocardial infarction (MI). Furthermore, the inhibition of casein kinase 2 (CK-2α1), an enzyme that activates HDAC2, exerted a beneficial effect with a reduction of cardiac hypertrophy (111). It is relevant to underline that, as described before, the functionality of all the epigenetic factors mentioned depends on cell metabolism.

## Sex-Specific Features of CAVD

There are growing evidences showing that there are sex-related differences in patients with CAVD in terms of clinical phenotypes (112), pathobiology, gene profiling (113, 114), and outcome (115). Although the overall incidence of CAVD is higher in men, the disease in women occurs at more advanced ages, which worsens the outcome of valve surgical substitution. This calls for sex-specific investigations in order to unravel the mechanisms responsible for this dimorphism and to ensure more effective treatment in the two sexes (116). Several reasons may account for these differences, such as the hormonal levels (117) and the generally protective role of feminine hormones (i.e., estrogen) against insurgence of atherosclerosis (118), or the higher mechanical load experienced by the leaflets in relationship with the bigger body surface area and cross-sectional area of the aortic annulus in men vs. women (119). Like for atherosclerosis, the protection of the female hormones appears to end with the menopause. In fact, studies demonstrated that valve calcification does not develop until after menopause in female subjects (120), or the end of estrogen replacement therapy (ERT) in post-menopausal women (121, 122). More at a molecular level, it is known that female hormones interfere with pathways involved in cardiovascular calcification. For example, Wu and colleagues found that estrogen reduces expression of HIF-1α and this reduces BMP-2 signaling and vascular calcification in rats (123). It was also reported that inhibition of androgen receptor in VSMCs protects from testosterone-induced VSMC calcification *in vitro* (124). On the other hand, testosterone has also been found protective against calcification, highlighting a complexity in the assessment of its role in different *in vitro* models (125).

Apart from the direct effect of hormones on calcification, sex-specific differences in the matrix remodeling and calcification programs were reported in male *vs*. female VICs. In particular, Authors demonstrated that male VICs formed calcific nodules with higher area and greater matrix remodeling with early calcific markers compared to female cells. Furthermore, β-estradiol treatment exerted an inhibitory effect only on female VICs (126). This might be explained by a different phenotypic composition of VICs in the two sexes, with male VICs more prone to calcification and lower responsivity to sex hormones, and female VICs more programmed for fibrotic remodeling of the valve leaflets (127). Although to our knowledge there have been no systematic studies addressing the possibility of sex-specific differences in phenotypic composition of VICs in the two sexes, such as for example by single cell RNA sequences, studies suggest that this might be the case. For example, *in vitro* studies demonstrated that female VICs, even under pro-calcific stimuli, have a lower calcification potential compared to male cells and this was linked to higher expression of MGP and BCL-2 (128). Furthermore, male VICs are characterized by an increased IL-6, BMP-2, and MMP-1 secretion, and a greater activation of HIF-1α pathway, suggesting an involvement of sex in metabolic control of valve calcification (128). Taken together, these data suggest that there is a close correlation between gender and pathophysiological programming of CAVD. Studies investigating in deeper details the molecular programming underlying these differences will be necessary to substantiate specific strategies to address the pathology in women *vs*. men.

## Conclusions

This review highlights the complex relationships between inflammation, epigenetic regulation, and metabolism alterations as a potential crucial factor in the onset and progression of CAVD. Although drugs with a potential to improve metabolism or restore a correct epigenetic setup represent a promising and novel therapeutic approach, a deeper understanding of the molecular pathways involved is still necessary, in order to improve patient management. Similarly, further investigations are needed to unravel the role of alternatives pathways in pathology development and progression in men and women. This will contribute in the identification of sex-tailored prognostic/diagnostic strategies and new therapeutic opportunities.

## Author Contributions

SF reviewed the literature, wrote the manuscript, and conceived the figures. MP revised the manuscript and the figures. All authors contributed to the article and approved the submitted version.

## Conflict of Interest

The authors declare that the research was conducted in the absence of any commercial or financial relationships that could be construed as a potential conflict of interest.

## Publisher's Note

All claims expressed in this article are solely those of the authors and do not necessarily represent those of their affiliated organizations, or those of the publisher, the editors and the reviewers. Any product that may be evaluated in this article, or claim that may be made by its manufacturer, is not guaranteed or endorsed by the publisher.
